# Monoclonal Antibodies against the MET/HGF Receptor and Its Ligand: Multitask Tools with Applications from Basic Research to Therapy

**DOI:** 10.3390/biomedicines2040359

**Published:** 2014-12-03

**Authors:** Maria Prat, Francesca Oltolina, Cristina Basilico

**Affiliations:** 1 Department of Health Sciences, Università del Piemonte Orientale, via Solaroli 17, 28100 Novara, Italy; E-Mail: francesca.oltolina@med.unipmn.it; 2 Laboratory of Exploratory Research, Candiolo Cancer Institute, Str. Prov. 142, 10060 Candiolo, Italy; E-Mail: cristina.basilico@ircc.it

**Keywords:** agonist monoclonal antibodies, antagonist monoclonal antibodies, tyrosine kinase receptor, tumor therapy

## Abstract

Monoclonal antibodies can be seen as valuable tools for many aspects of basic as well as applied sciences. In the case of MET/HGFR, they allowed the identification of truncated isoforms of the receptor, as well as the dissection of different epitopes, establishing structure–function relationships. Antibodies directed against MET extracellular domain were found to be full or partial receptor agonists or antagonists. The agonists can mimic the effects of the different isoforms of the natural ligand, but with the advantage of being more stable than the latter. Thus, some agonist antibodies promote all the biological responses triggered by MET activation, including motility, proliferation, morphogenesis, and protection from apoptosis, while others can induce only a migratory response. On the other hand, antagonists can inhibit MET-driven biological functions either by competing with the ligand or by removing the receptor from the cell surface. Since MET/HGFR is often over-expressed and/or aberrantly activated in tumors, monoclonal antibodies can be used as probes for MET detection or as “bullets” to target MET-expressing tumor cells, thus pointing to their use in diagnosis and therapy.

## 1. Introduction

Monoclonal antibodies (mAbs) have been revealed to be extremely useful reagents, because of their high specificity, affinity, and robust structure. Moreover, because of their modular structure they can be easily engineered through molecular biology technologies; this is extremely useful in case of a therapeutic use in humans [1]. One of the best examples in cancer therapy is Trastuzumab (Herceptin^®^: Genentech Inc.), which was approved by the FDA in 2006 for patients with invasive breast cancers over-expressing the tyrosine kinase orphan receptor HER2, generally in a combined therapy with chemotherapeutics [2]. More recently (2010), its use was also approved for patients with HER2-over-expressing Metastatic Gastric or Gastroesophageal (GE) Junction Adenocarcinoma (http://www.cancer.gov/cancertopics/druginfo/fda-trastuzumab).

MET, the tyrosine kinase receptor of HGF, is another potential target in different types of solid and hematological neoplasms, such as colorectal carcinoma [3], glioblastoma [4,5], and breast carcinoma [6].

MET and its physiological ligand, hepatocyte growth factor/scatter factor (HGF), were discovered in the 1980s as a result of three independent lines of research, reflecting the pleiotropism of this receptor/ligand couple. MET was first identified in a rearranged tumorigenic form, giving rise to the TPR-MET fusion protein [7], and opening to the discovery of the full-length proto-oncogene [8]. Other research groups identified a molecule performing as a hepatocyte growth factor [9,10] or as a scatter factor [11], *i.e.*, promoting epithelial mesenchymal transition, which was then found to be the same molecule [12,13] and the ligand of the MET receptor [14,15]. The two activities, mitogenic and motogenic, can be combined in so-called invasive growth [16], which contributes to organ development during embryogenesis [17], and to tissue maintenance and repair in adults [18]. Moreover, like many other growth factors, HGF is also a survival factor [19,20]. The many different cellular responses observed upon HGF-dependent MET stimulation are the consequence of the activation of distinct signaling pathways downstream of MET, which can intersect and also cooperate with other signaling pathways, and can differ depending on the histotype and developmental stage of the responding cells [16,21,22].

The same biological responses that are strictly regulated by HGF in MET-expressing cells in normal or repairing physiological settings can be subverted and become responsible for tumor development and metastasis [21,23]. Abnormal activation of the receptor, leading to neoplastic transformation and tumorigenesis, can occur through different mechanisms such as point mutation [24] or over-expression as consequence of either gene amplification [25] or post-transcriptional dysregulation [21,26,27]. Moreover, the establishment of ligand-dependent autocrine or paracrine loops may play a relevant role in abnormal MET activation; this mechanism also appears to be a requirement in the case of MET mutation or receptor over-expression [28,29]. MET can also be activated in an HGF-independent way upon interaction with other cell surface receptors, such as semaphorin-activated plexins [30], other ligand-activated tyrosine kinase receptors, e.g., EGFR [31] and IGFR-1 [32], cell surface adhesion molecules, e.g., the α6β4 integrin [33], or some variants of CD44 [34]. These multireceptor platforms involving MET display higher efficiency in the recruitment of signal transducers, thus leading to MET-mediated signal amplification and representing an alternative way to stimulate MET-driven biological responses [23].

While in the case of some tumors MET-mediated cellular activities may be independent from the ligand, all the physiological activities mediated by this receptor are strictly dependent on and regulated by HGF stimulation. Thus, depending on the target system considered, MET activation may trigger detrimental responses (uncontrolled proliferation and tissue invasion in cancer) or beneficial effects (tissue and organ development, maintenance, and repair). Antibodies are reagents of high specificity and affinity and can be viewed as alternative ligands of the receptors against which they were raised, besides being valuable probes for antigen identification. Antibodies may act as agonists, thus mimicking the natural ligands and promoting useful biological responses—for example, stimulating the survival and proliferation of cells in injured tissues. Conversely, they may behave as antagonists, inhibiting MET-mediated uncontrolled excessive responses of tumor cells. In the latter scenario, antibodies against the ligand could represent valuable tools for hampering the ligand–receptor interaction.

Herein we review the properties of monoclonal antibodies directed against the HGF/MET receptor couple and their possible applications ranging from basic research to therapy. A brief summary of the structures of the two partners, instrumental for elucidating their interaction with the mAbs, is also presented.

## 2. HGF and MET: Structural Properties, Interaction and Receptor Activation

**Figure 1:**
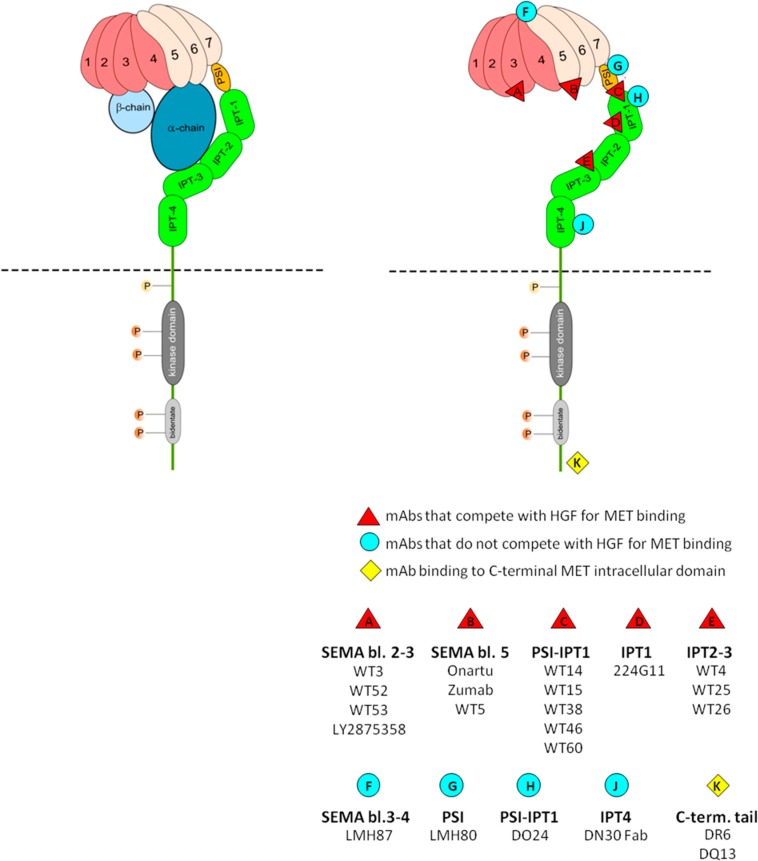
Schematic representation of MET interactions with HGF or with anti-MET antibodies. **Left**: Hypothetical model of HGF/MET interactions. The mature form of HGF consists of an α (dark blue) and a β (light blue) chain held together by a disulphide bond. MET is a single-pass, multi-domain, disulphide-linked α/β heterodimer. Its extracellular portion consists of three domains: the SEMA domain, folded into a seven-bladed β propeller, which encompasses the whole α chain (dark pink) and part of the β-subunit (light pink); the cysteine-rich PSI domain (orange); and the four immunoglobulin-like IPT domains (green). The intracellular region consists of the kinase domain (dark grey) and a multifunctional docking site (bidentate, light grey). In this model, based on data from different independent studies [38,39,40,41,42,43], the α chain of HGF interacts on one side with blades 4–6 of the SEMA β-propeller and on the other with the IPT 2–3 regions, while the β chain interacts with blades 2–3 of the SEMA β propeller; **Right**: Epitope mapping of anti-MET antibodies. Antibodies directed against the MET ectodomain (ECD) recognize epitopes localized in many different areas of the receptor.

HGF is a multi-domain heterodimeric protein, consisting of two α and β subunits linked by a disulphide bond (Figure 1). The α chain is composed of an *N*-terminal hairpin segment followed by four disulphide-bond-stabilized kringle domains, while the β chain has the structure of a serine protease, but, due to the substitution of three amino acids, is devoid of enzymatic activity. HGF is secreted as an inactive single-chain precursor, which can be stored in tissues through high affinity binding to proteoglycans. In response to tissue injury, pro-HGF is then activated through proteolytic cleavage, to induce a local and transient burst of active HGF that promotes tissue regeneration [35].

MET receptor is a single-pass transmembrane heterodimeric tyrosine kinase glycoprotein, composed of two polypeptide subunits linked by disulphide bonds and consisting of different structural and functional domains (Figure 1). The extracellular region is responsible for ligand binding and consists of three functional domains: the Sema domain, structured as a seven-bladed propeller and encompassing the whole α-subunit of the receptor and part of the β-subunit (amino acids 1–518); the cysteine-rich PSI domain (amino acids 519–561, with high homology to plexin, semaphoring, and integrin); and four immunoglobulin-like IPT domains (amino acids 562–932). The intracellular region contains the kinase domain responsible for receptor activation through transphosphorylation, some regulatory sequences, and a multifunctional docking site which, once phosphorylated, is able to recruit signal transducers, effector molecules and adaptors (see reviews [21,23,36]. Mature MET is cleaved from its single chain precursor into its two subunits by the furin protease in the Golgi apparatus [37].

The interaction between MET and its ligand HGF has not been fully elucidated. It appears to be quite complicated and to involve different binding sites on both molecules. The crystal structure of the HGF β-chain, in complex with the SEMA–PSI domain of MET ectodomain (ECD) [38], unveiled the presence of contacts between the bottom face of blades 2 and 3 of the SEMA β-propeller and the β-chain of HGF. This is a low affinity interaction, but is mandatory for ligand-induced MET activation and requires a proteolitically-cleaved active HGF β-chain conformation [39]. However, no crystal structure elucidating the interface between MET ECD and HGF α-chain, responsible for the high-affinity binding of the two partners, is available so far. Various studies suggested the involvement of either the SEMA domain [40,44] or the IPT region [41] as MET binding site for the HGF α-chain. Recently, hints to reveal this elusive interaction came from the detailed epitope mapping of HGF-displacing anti-MET antibodies. The crystal structure of a ternary complex including the MET SEMA–PSI, the HGF β-chain, and the Fab of onartuzumab (see the MET antagonist antibody section) disclosed that this antibody binds to blades 4–6 of the SEMA β-propeller [42], and competes with full size HGF for binding to MET. Since onartuzumab binds to the MET SEMA–PSI simultaneously with the HGF β-chain, it must interfere with the binding of HGF α-chain to the receptor. Data derived from the epitope mapping of a panel of HGF-displacing anti-MET antibodies identified four separate hotspots throughout the extracellular region of MET [43]. One hotspot is located within SEMA blades 2 and 3 and corresponds to the HGF-β binding site; the same epitope is also recognized by another MET-antagonist/HGF-displacing antibody, LY2875358 [45]. A second hotspot lies in SEMA blade 5 and overlaps with the onartuzumab binding site [42], while the third localizes within IPT domains 2–3, a region of MET previously proposed to contain a binding site for HGF α-chain [41]. Finally, some antibodies bind to a formerly unidentified epitope, which is located between the PSI and IPT-1 domains, and could provide a new site of MET/HGF interaction. All this information, together with the results obtained in antibody competition experiments, suggests that HGF binds to multiple sites within the MET ECD. A possible scenario is presented in Figure 1. While HGF-β binds to blades 2 and 3 of the SEMA β-propeller, the HGF α-chain seems to interact with the SEMA blade 5 on one side and with the IPT domains 2–3 on the other. According to this hypothesis, the PSI would act as a hinge, allowing the SEMA and IPT domains to bend one over the other (in a way similar to what is proposed for plexins) [46]. This would lead to the creation of a pocket into which the HGF α-chain fits.

Experiments performed with mAbs clearly showed that receptor activation requires its dimerization, since it is strictly dependent on antibody bivalence. Indeed, monovalent Fab could not induce receptor transphosphorylation, which was recovered upon the addition of a secondary bivalent antibody [47]. This exemplified proof of concept was then exploited to produce monovalent antibodies with inhibitory activity for cancer cells [4,48]. Full activation, *i.e.*, dimerization-induced transphosphorylation of the receptor, is a requirement for some MET-mediated biological responses, such as cell proliferation [47]. Similar conclusions were reached with experiments performed by introducing mutations in the two natural splice variants of HGF, NK1 and NK2. NK1, which is a MET agonist, forms a head-to-tail dimer complex in crystal structures, and mutations within the NK1 dimer interface convert it into a MET antagonist. In the mirror situation, mutations disrupting the close configuration of NK2, which is basally a MET antagonist, convert NK2 to a receptor agonist, but reintroduction of mutations disrupting the NK1/NK1 dimer interface converted back to an inactive ligand [49].

After receptor activation, *i.e.*, phosphorylation on Y_1230_, Y_1234_, and Y_1235_, two other tyrosines (Y_1349_ and Y_1356_) embedded within a degenerate consensus sequence are phosphorylated and act as a double docking site for recruitment of both transducers and adaptors. This leads to the activation of multiple intracellular signaling pathways, the most important of which are the Ras→MAPK ERK1/2 and the PI 3 kinase→Akt pathways [21,23,36] (An up-to-date and complete list is out the scope of this review, since it is already present in this issue [22]).

## 3. mAbs Recognizing the HGF/MET Receptor

Depending on the desired antibody specificity, immunization was performed with different strategies. While mAbs targeting MET ectodomain were produced upon immunization with cells over-expressing the receptor [50,51], or with purified proteins including a MET ectodomain-Fc hybrid molecule [52], the isolated SEMA domain [46] or the α-chain [51], mAbs recognizing the intracellular domain of MET were produced by immunizing with the *C*-terminal 19 amino acids coupled to a carrier protein [53,54]. Peptides were also used as immunogens to prepare polyclonal antibodies against the *C*-terminal sequence, as well as antibodies against differentially phosphorylated forms of the MET protein [55]. Most of the antibodies are murine mAbs, and most of them were then humanized using recombinant technology in view of their therapeutic use. Fully human monoclonal antibodies have been generated using the Xeno-Mouse technology [56]. Recombinant antibodies such as scFv from a human naïve library [57] and, more recently, chimeric llama–human antibodies [43] have been reported. On this basis it is possible to conclude that, while linear peptide sequences can be used to raise antibodies against the intracellular domains, only full size domains in which the native protein conformation is preserved are efficient immunogens in the case of the receptor ectodomain. In consequence, most of the antibodies with the latter specificity preferentially recognize receptors in their native conformation.

## 4. mAbs as Probes to Identify the Receptor

Since by definition monoclonal antibodies are reagents of high specificity and affinity, from the beginning they have been used as probes to analyze the expression of MET on tumors and normal tissues [27,53]. Briefly, this receptor was found to be over-expressed in a variety of cancers, especially of epithelial origin [28,54,58,59,60,61]. However, tumors of mesenchymal origin, such as osteosarcoma and musculoskeletal tumors, also over-express MET [62,63], and recently MET expression was reported to be useful for the classification of neuroglioblastoma subtypes [64]. For a complete and up-to-date list of MET expression in tumors see www.vai.org/met.

Historically, the first mAbs directed against the extracellular domain of MET, in combination with those against the intracellular *C*-terminal peptide, contributed to identify *C*-terminally truncated receptor isoforms, as well as to elucidate their origin and biosynthetic pathways. Indeed, together with the full size 190 kDa MET protein, these antibodies immunoprecipitated two additional MET proteins of 140 and 130 kDa [50]. The first protein (p140^MET^), which is associated to the plasma membrane through its β chain, consists of a 50 kDa α chain and an 85 kDa β chain, which is truncated in its intracellular domain. The second protein (p130^MET^) is a soluble receptor isoform, which is released in the culture supernatant and consists of a 50 kDa α chain linked to a 75 kDa fragment of the β chain. Both truncated forms lack the tyrosine kinase domain, are basally produced from post-translational proteolysis [53,65], and may represent a safety mechanism aimed at preventing ligand-independent intracellular activation of the HGF receptor, attenuating MET signaling in steady-state conditions [65]. Moreover, the production of the soluble form, which is released by shedding catalyzed by ADAM metalloproteases [66,67], can be enhanced by binding of mAbs against the MET ectodomain, as well as by other agents, such as phorbol esters, suramin, and lysophosphatidic acid [53,68,69]. In this way, not only is the number of receptor molecules on the cell surface decreased, but a decoy moiety able to interact with both HGF (sequestering and antagonizing the ligand) and the full-length MET (impairing dimerization and transactivation of the native receptor) is also generated, thus strengthening MET signaling inhibition [70,71]. This concept is now being pursued as a strategy for cancer therapy through the use of a monoclonal antibody (see further information in the antagonist MET antibodies section).

The antibodies against the MET ectodomain were also useful in elucidating the localization of the receptor in polarized epithelia. MET was found to be concentrated around the cell–cell contact zone, with a distribution pattern overlapping that of the cell adhesion molecule *E*-cadherin and selectively exposed at the basolateral plasma membrane domain, where it is delivered directly from the Golgi apparatus during its synthesis [65]. Indeed, this localization is the most appropriate to favor the paracrine interaction with the ligand, which is generally produced by tissues of mesenchymal origin underlying epithelial tissues.

In the era of targeted therapy, the eligibility of patients for a specific treatment depends on the presence of a precise genetic lesion and a molecular diagnosis is mandatory. In this context, MET antibodies may represent valuable diagnostic tools for immunohistochemistry and for use with non-invasive technologies such as PET and SPECT-Scan. Interestingly, the DN-30 anti-MET antibody (see next paragraph) labeled with the positron-emitting isotope ^89^Zr can be used for imaging of MET-expressing tumors [72].

## 5. Agonist MET mAbs

Many reports from the literature show that mAbs directed against the ectodomain of tyrosine kinase receptors can activate the receptor and trigger biological responses [73,74,75,76]. This has been extremely useful in studying the biochemical and biological responses deriving from the activation of the so-called orphan transmembrane tyrosine kinase receptors, for which no ligand has been identified so far [77,78,79].

In the case of MET, antibodies directed against the ectodomain allowed for dissection of the different biological responses and the correlated transduction pathways.

Two antibodies (DO-24 and DN-31 [50]) that behave as reciprocal cross-competitors for binding to the receptor were found to act as full agonists, being able to trigger all the biological effects elicited by the natural ligand HGF, e.g., motility, proliferation, cell survival, invasion, tubulogenesis, and *in vivo* angiogenesis [47,80]. Two other mAbs (DN-30 and DL-21) that behave as partial agonists and bind to different epitopes of the receptor were able to activate only motility and protection from apoptosis [47,81,82]. All the antibodies were able to trigger receptor phosphorylation, which was found to be strictly dependent on mAb bivalence; in fact, the monovalent Fab was ineffective, and activation was recovered by the addition of a secondary anti-mouse Ig antibody [47]. Only the full agonist mAbs were found to be able to induce and sustain the expression of urokinase-type plasminogen activator (uPA) receptor for prolonged periods of time [47]. By binding uPA at the cell surface, this receptor focalizes there a proteolytic machinery, which can recruit and activate metalloproteases with potent extracellular matrix-degrading action. This activity plays a key role in invasive growth, a distinguished feature of the HGF/MET axis, which combines proliferation and migration and is particularly important in tubulogenesis. Using the two classes of agonist mAbs, the dissection of the two groups of biological responses, previously analyzed in canine epithelial cells, was confirmed also for Kaposi sarcoma cells [83]. In this case, the partial agonism of the mAbs correlated with a reduced and short ERK-1/2 activation, compared with that achieved by full agonist mAbs, while in the case of other transducers or adaptors—PI 3kinase, JNK and Gab-1—no differences were detected. Thus the PI 3 kinase–Akt pathway is also fully activated by partial agonist mAbs, which can elicit motogenicity and protection by apoptosis. The epitopes recognized by the mAbs DO-24 and DN-30 have been localized outside the HGF binding site, since they do not compete with the natural ligand. In particular, the DN-30 mAb binds in the IPT-4 region, while the DO-24 mAb binds around the PSI-IPT-1. While both mAbs induce receptor activation, because of their bivalence, only DO-24 is a full agonist promoting all MET-mediated biological responses. It follows that simple MET dimerization is not enough for full receptor activation, for which further requirements need to be met, which may be linked to the particular epitope recognized by the antibody. It is worth noting that the epitope recognized by DO-24 overlaps with the primary binding site of the *L. monocytogenes* Internal B protein, which activates the MET receptor and promotes the bacterial invasion of the host cells, as identified by cross-inhibition experiments [84] and co-crystallization of the MET ectodomain with Internalin B [85].

The DN-30 mAb is a partial MET agonist, but also behaves as an antagonist, and has been further developed as a monovalent antibody for anti-cancer therapy (see Antagonist MET mAbs section). The different contrasting activities of the bivalent form may be linked to the amount of mAbs used in the different experimental settings; indeed, the agonistic activity is generally more pronounced at low doses, and disappears at higher doses [30]. The fact that the same mAbs can behave as partial agonist and antagonist was observed also for Trastuzumab [86].

The agonist mAbs were able to protect cardiomyoblasts from apoptosis induced by oxidative stress or by hypoxia induced by cobalt chloride treatment [81,82]. They also counteracted apoptosis, as analyzed by different parameters such as DNA fragmentation, cell shrinkage, annexin V positivity, mitochondrial translocation of bax, caspase activation, and nuclear aspect. Protection from apoptosis was dependent on an active MET, since it could be inhibited by treatment of cardiomyoblasts with MET-specific si-RNA or by the MET tyrosine kinase inhibitor PHA-665752. MET agonist antibodies proved to be effective in inhibiting autophagy as well, a less considered mechanism of cell damage in heart diseases. Indeed, it is acknowledged that basal levels of autophagy are required for cardiac homoeostasis, since cardiomyocytes are long-living cells and autophagy allows the removal of damaged molecules and organelles [87]. However, autophagy can act as a double-edged sword in the cardiovascular system and indeed an autophagic flux, with the involvement of the Beclin p62, LC3, was triggered in response to ischemia/reperfusion injury, which thus resulted in detriment to the cells [82,88,89]. The protection from autophagy afforded by the agonist mAbs, as well as by the natural ligand, was mTOR dependent, since it was prevented by the specific mTOR inhibitor Temsirolimus [82]. MET agonist mAbs were also able to trigger motility of cardiomyocytes, as analyzed in wound healing and in a Boyden chamber assay. It is worth noting that both activities relay in a PI 3 kinase→Akt pathway, which indeed both full and partial agonists can activate [47]. These data thus validate these anti-MET mAbs being valuable substitutes of the natural ligand HGF, with the added advantage of being more easily obtained in a biologically active, highly stable, and purified form.

## 6. Antagonist MET mAbs

The development of antagonist antibodies directed against MET has been hindered by the intrinsic bivalent nature of antibodies. In fact, MET activation is a consequence of the approach of two or more receptors that transphosphorylate each other and start the signal transduction cascade. This early event is prompted mainly by the interaction of MET with its ligand HGF or by the physical proximity of receptors at the cell membrane due to MET over-expression. Binding of a bivalent antibody to the extracellular region of MET may induce receptor homodimerization and subsequent downstream activation, thus generating an agonistic rather than an antagonistic outcome. This problem may be circumvented by engineering monovalent antibodies devoid of ligand-mimetic properties.

It is not the case that the most advanced MET antibody in clinical evaluation, onartuzumab [42], is the humanized, affinity-maturated monovalent form of the 5D5 antibody [90]; that was genetically engineered by the knob-into-hole technology to produce a one-armed monovalent MET antibody. In this way, a potent agonistic antibody, 5D5, has been turned into a pure antagonist of MET signaling. The presence of an intact Fc domain allows binding to the neonatal Fc receptor (FcRn), an important function for the long half-life of antibodies *in vivo*, thus providing onartuzumab with good pharmacokinetic properties. Onartuzumab acts by competing with HGF, in particular with the NK1 portion of the HGF α-chain, for binding to MET. Upon crystallization of a ternary complex composed of the antibody in the Fab format, the SEMA–PSI domain of MET, and the β-chain of HGF, the exact amino acid residues of MET involved in onartuzumab binding have been identified. They are located within blades 4–6 of SEMA β-propeller and overlap with the secondary binding site of the *Listeria monocytogenes* protein Internalin B. This antibody inhibited tumor growth in HGF-dependent preclinical models of glioblastomas [4] and pancreatic carcinomas [91], and showed good tolerability and satisfactory pharmacokinetic properties in Phase I studies [92]. Phase II studies evaluating onartuzumab in combination with chemotherapy and/or other targeting agents are currently ongoing (see Table 1). The final results of a Phase II trial showed that patients with advanced non-small cell lung cancer bearing tumors with high MET expression (scored as “MET positive”) benefit from the combination of onartuzumab and erlotinib (an inhibitor of EGF receptors) [56]. However, the efficacy results observed in this study were not confirmed in the subsequent large Phase III MetLung trial (NCT01456325, see Table 1), which was stopped due to futility, since the addition of onartuzumab to erlotinib did not improve overall survival (OS) or overall response rates [93]. Two additional Phase III trials of onartuzumab and erlotinib in MET-positive NSCL patients started last year, while the Phase III MetGastric trial is testing the activity of onartuzumab in combination with chemotherapy in Her2-negative, MET-positive gastroesophageal cancer patients (see Table 1).

**Table 1:** Clinical trials involving anti-MET antibodies.

Antibody	Clinical Trials		
	Phase	Study No.	Purpose
**Onartuzumab**	Phase I	NCT01974258	evaluation of the maximum tolerated dose and dose-limiting toxicities of vemurafenib and/or cobimetinib when used with onartuzumab in cancer patients
	Phase I	NCT02031731	examination of the pharmacokinetics and safety of Onartuzumab (MetMAb) in chinese patients with locally advanced or metastatic solid tumors
	Phase I/II	NCT02044601	goal of the Phase I part: to find the highest tolerable dose of onartuzumab that can be given with erlotinib and standard chemoradiation (paclitaxel and carboplatin) to patients with NSCLC
			goal of the Phase II part: to learn if onartuzumab plus erlotinib and chemoradiation can help to control NSCLC
	Phase I	NCT01897038	evaluation of the maximum tolerated dose (MTD) and dose-limiting toxicities of onartuzumab as single agent and in combination with sorafenib in patients with advanced hepatocellular carcinoma
	Phase II	NCT01632228	evaluation of the safety and efficacy of onartuzumab (MetMAb) in combination with bevacizumab as compared to bevacizumab alone and to onartuzumab as monotherapy in patients with recurrent glioblastoma
	Phase II	NCT01590719	evaluation of the efficacy and safety of onartuzumab (MetMAb) in combination with mFOLFOX6 in patients with metastatic HER2-negative adenocarcinoma of the stomach or gastroesophageal junction
	Phase II	NCT01519804	evaluation of the efficacy and safety of onartuzumab (MetMAb) in combination with paclitaxel plus platinum in patients with incurable Stage IIIB or Stage IV squamous non-small cell lung cancer (NSCLC)
	Phase II	NCT01418222	evaluation of the efficacy and safety of FOLFOX/bevacizumab with onartuzumab (MetMAb) versus placebo as first-line treatment in patients with metastatic colorectal cancer
	Phase II	NCT01186991	estimation of the efficacy and evaluation of the safety and tolerability of MetMAb + bevacizumab + paclitaxel and MetMAb + placebo + paclitaxel versus placebo + bevacizumab + paclitaxel in patients with metastatic or locally recurrent, triple-negative breast cancer who either have not received treatment (first line) or have progressed after one conventional cytotoxic chemotherapy regimen (second line)
	Phase II	NCT01496742	evaluation of the efficacy and safety of RO5490258 (MetMab) in combination with either of two backbone chemotherapy regimens in the first line setting in patients with incurable Stage IIIB or IV non-squamous non-small cell lung cancer
	Phase III	NCT02031744	evaluation of the safety and efficacy of MetMAb (onartuzumab) in combination with Tarceva (erlotinib) compared with treatment with Tarceva alone in patients with incurable Met-positive non-small cell lung cancer (NSCLC)
	Phase III	NCT01887886	evaluation of the safety and efficacy of onartuzumab in combination with erlotinib in patients with previously untreated, unresectable stage IIIB or IV non-small cell lung cancer identified to carry an activating EGFR mutation and MET-positive
**Onartuzumab**	Phase III	NCT01662869 (MetGastric)	evaluation of the efficacy and safety of onartuzumab (MetMAb) in combination with mFOLFOX6 in patients with metastatic HER2-negative and Met-positive adenocarcinoma of the stomach or gastroesophageal junction
	Phase III	NCT01456325 (MetLung)	evaluation of the efficacy and safety of onartuzumab (MetMAb) in combination with Tarceva (erlotinib) in patients with incurable non-small cell lung cancer identified to be Met diagnostic-positive
**H224G11/ABT700**	Phase I	NCT01472016	evaluation of the safety, pharmacokinetics (PK), and preliminary efficacy of ABT-700 in subjects with advanced solid tumors that may have MET amplification or c-Met over-expression
**LY2875358**	Phase I	NCT01287546	evaluation of a recommended Phase II dose range of LY2875358 that may be safely administered to patients with advanced cancer
	Phase I	NCT02082210	evaluation of a recommended schedule and dose range for LY2875358 when given with ramucirumab (an anti-VEGFR2 antibody) in cancer patients
	Phase I	NCT01602289	assessement of the safety and tolerability of LY2875358 as monotherapy or in combination with erlotinib or gefitinib in Japanese patients with advanced or metastatic cancer
	Phase II	NCT01900652	evaluation of the efficacy of LY2875358, administered alone or in combination with Erlotinib, in MET-positive NSCL cancer patients that experienced a disease progression during Erlotinib treatment
	Phase II	NCT01897480	comparison of the efficacy of LY2875358 plus erlotinib versus erlotinib alone in NSCL cancer patients that advanced to Stage IV. All participants will get erlotinib alone, for approximately 8 weeks. Patients with radiographic disease control at the end of the erlotinib lead-in study period will be randomly assigned to receive LY2875358 plus erlotinib or erlotinib alone
	Phase II	NCT01874938	evaluation of the effectiveness of LY2875358 in MET-positive advanced gastric or gastroesophageal junction (GEJ) cancer patients
**ARGX-111**	Phase I	NCT02055066	evaluation of the dose-limiting toxicity and of the pharmacokinetic profile of ARGFX-111 in patients with MET-over-expressing advanced cancer

A similar strategy was pursued in the case of the bivalent antibody DN-30 [47], which acted as a partial agonist of MET: a monovalent Fab form of the antibody was engineered [70] that maintained the sub-nanomolar affinity for MET. This monovalent Fab, however, is unable to induce receptor homodimerization and kinase activation and therefore behaves as a pure antagonist. DN-30 Fab binds to the IPT-4 domain of MET and promotes receptor shedding: upon antibody binding, the extracellular portion of MET is proteolytically cleaved close to the cell membrane, causing the release of a soluble receptor in the extracellular space [94], while the intracellular portion is rapidly degraded by the proteasome [66]. Therefore, ectodomain shedding results in the generation of a soluble “decoy” MET that may on one side bind to HGF, sequestering it from the environment, and may on the other side form catalytically-inactive heterodimers with full-length transmembrane receptors that survived cleavage [71]. As a consequence, MET downstream signaling is abrogated and MET-mediated biological activities are strongly impaired. DN-30 Fab (also called monovalent DN-30, MvDN30) reduces the anchorage-independent growth of a broad panel of HGF-dependent or -independent tumor cells [70], and induces growth arrest and apoptosis of tumor cells addicted to MET signaling in *in vitro* proliferation assays. In preclinical models of MET-driven human gastric and lung carcinoma [70,95], as well as glioblastoma [48], DN-30 Fab treatment was effective in delaying tumor growth. Thanks to its peculiar mechanism of action, DN-30 Fab works in settings of both ligand-dependent and -independent MET activation, and therefore represents a very appealing therapeutic tool. However, the clinical application of an antibody Fab fragment is hindered by its short half-life in plasma due to high kidney clearance. To overcome this drawback, DN-30 Fab has been conjugated with polyethylene glycol to obtain a stabilized molecule with improved *in vivo* activity [70]. Recently, an innovative gene therapy approach has been proposed to bypass Fab’s short half-life. DN-30 *Fab* gene delivery allows continuous and sustained production of the therapeutic molecule by the host, and gave encouraging results in two preclinical models of MET-driven cancers [48,95]. Gene therapy may therefore represent an ideal delivery system in the case of Fabs and other antibody fragments. Other strategies for DN-30 Fab implementation are currently under investigation, including genetic engineering of the antibody to incorporate albumin-binding domains or supplementary immunoglobulin domains for enhancing Fab stability or to generate recombinant fusion proteins for a multi-targeting approach (personal communication).

Humanized anti-MET nanobodies represent another approach to the generation of monovalent antibody-derived molecules able to bypass the risk of inducing an agonistic response. Nanobodies derive from heavy chain antibodies, discovered in Camelidae in the early 1990s. The Camelidae humoral immune response is peculiar: In the serum of these animals conventional antibodies coexist with the so-called heavy chain antibodies (HCAbs), functional heavy chain homodimers that do not associate with a light chain and lack the first heavy chain domain [96]. Therefore, HCAbs consist of a single variable domain (VHH) and two constant domains (CH_2_ and CH_3_). The VHH domain corresponds to the structural and functional domain of the Fab fragment of conventional antibodies. Nanobodies (also called single domain antibodies, sdABs) are isolated recombinant VHHs that retain the full antigen-binding capacity of the original HCAb. An anti-MET nanobody was generated by combining two different building blocks, one targeting MET and the other binding human serum albumin, for half-life extension. It binds with high affinity to MET and displays *in vitro* inhibitory activity on human myeloma cells harboring a MET-HGF autocrine loop [97].

In recent years, a number of bivalent MET-targeting antibodies have been developed, and some of them are currently under clinical investigation. Many efforts have been made to identify full-length antibodies combining strong inhibitory properties with a negligible agonistic activity. In fact, the importance of antibody effector functions has progressively emerged, and antibody-dependent cytotoxicity (ADCC) especially has been demonstrated to play a major role in antibody efficacy [98]. Cytotoxic effector functions are governed by the antibody Fc domain, and cannot therefore be elicited by Fab fragments or nanobodies; similarly, onartuzumab, though possessing an intact Fc domain, is devoid of antibody-dependent cytotoxicity (ADCC) or complement-dependent cytotoxicity (CDC) being produced in *E. coli* and therefore aglycosylated [42].

R13 and R28 [99] are two fully human MET antibodies that need to be combined to interfere with HGF for binding and to elicit ADCC. According to their predicted mechanism of action, the first antibody locks the receptor in an inactive state and facilitates binding of the second one, thus blocking HGF binding and avoiding MET activation.

A panel of antibodies (designated LMH) was generated by mice immunization with isolated MET SEMA domain or MET-expressing cells [51]. Their mechanism of action is poorly characterized; some of these antibodies seem to promote MET internalization and degradation, and/or to interfere with receptor recycling to the cell surface. The epitopes recognized by these antibodies have been mapped in detail. LMH83 and LMH85 do not bind to any linear peptides, indicating that their epitopes are conformational in nature. Four antibodies (LMH86–89) recognize an identical epitope localized between blades 3 and 4 of the SEMA β-propeller, while LMH82 and LMH84 bind, respectively, to blade 2 and blade 3. LMH80 is the only antibody of this panel mapping outside the MET α-chain, binding an epitope within the PSI domain.

CE-355621 derives from a panel of human monoclonal antibodies generated by immunizing XenoMouse transgenic mice with a fusion protein between the extracellular portion of MET and the Fc domain of human IgG (MET ECD-Fc), or with NIH-3T3 cells over-expressing human MET [52]. These antibodies display minimal agonistic activity, acting primarily as MET antagonists. CE-355621 inhibits MET activation by competing with HGF for binding to the receptor, although the detection of a modest decrease in total MET levels also suggests a possible induction of receptor internalization and degradation. *In vitro*, it displayed inhibitory properties in ligand-dependent biochemical and biological assays. *In vivo*, it delayed the growth of U87-MG human glioblastoma cells, which express both MET and HGF. Surprisingly, CE-355621 also exhibited robust inhibitory activity against GTL16 tumors, in which MET is constitutively phosphorylated due to gene amplification and receptor over-expression. Further investigations are required to elucidate the mechanism of action of this antibody in the case of HGF-independent MET activation.

SAIT301 is a humanized antibody that promotes MET degradation by the LRIG-mediated lysosomal pathway, which does not require receptor activation for its function [100]. SAIT301-induced MET down-regulation is therefore ubiquitin-independent and distinct from the Cbl pathway. Consistent with this hypothesis, SAIT301 displays minimal agonistic activity. Interestingly, this antibody substantially reduced the clonogenic growth of patient-derived cetuximab-resistant cancer cells, suggesting a potential role in overcoming resistance to EGFR inhibitors. Recently, the ability of SAIT301 to inhibit HGF-induced invasion and migration of nasopharingeal cancer cell lines has been described, suggesting a potential use for MET inhibitors in the treatment of this highly invasive and metastatic type of cancer [101].

A series of 21 antibodies—termed Specifically Engaging Extracellular MET (seeMET)—were generated by mice immunization with a purified MET α-chain [102]. They bind to 10 different epitopes within the MET α-chain and may represent valuable tools for diagnostics.

H224G11/ABT700 and LY2875358 are the first two bivalent anti-MET antibodies that entered clinical testing. H224G11/ABT700 is a humanized IgG1 antibody endowed with a fully antagonist activity [103], which recognizes an epitope within the IPT-1 domain of the MET ECD [43]. It blocks HGF binding and induces MET down-regulation by promoting receptor internalization and degradation. It inhibits proliferation, migration, and invasion of different cell lines. In addition to its direct effect on MET modulation, H224G11/ABT700 also triggers effector functions, exhibiting significant ADCC activity *in vitro*. In MET-dependent preclinical models, it inhibited the growth of both HGF-activated and constitutively phosphorylated tumor cell lines. In a Phase I clinical trial currently ongoing in subjects with advanced solid tumors, it is well tolerated and has demonstrated promising anti-tumor activity in patients carrying the MET gene amplification [104].

The humanized IgG4 antibody LY2875358 competes with HGF for binding to MET, and induces receptor internalization and degradation [45]. It recognizes an epitope within blades 2 and 3 of the SEMA β-propeller that overlaps exactly with the HGF-β binding site in MET ECD, and exhibits no functional agonistic activity. This antibody inhibits the growth and invasion of different tumor cell lines, and shows a potent anti-tumor effect in *in vivo* models of both HGF-dependent and HGF-independent MET activation, including a MET-amplified lung cancer xenopatient. LY2875358 has completed a Phase I dose escalation study in patients with advanced solid tumors [104] and is undergoing Phase II studies in combination with erlotinib in non-small cell lung patients; it is also being evaluated in a Phase II trial in MET-positive advanced gastric cancer patients (see Table 1).

Recently, another panel of bivalent antibodies binding MET with high affinity has been characterized [43]. These antibodies were generated by active immunization of llamas with MET-over-expressing human cells (MKN-45), and selected for their ability to compete with HGF. Here, as was not the case with nanobodies, the llama conventional antibody repertoire was exploited, due first of all to its extraordinary degree of homology with that of humans: in fact, the variable (V) regions of conventional llama antibodies are naturally encoded by the complete repertoire of V genes found in humans, and they can be humanized by few point mutations. Furthermore, immunization of outbred animals, like llamas, allows the generation of antibodies characterized by higher functional diversity than immunization of inbred mice. Antibody selection was performed using the phage display technology: the variable regions of llama antibodies were amplified, subcloned in a phagemid vector, and produced as Fab fragments fused to a phage envelope protein. The Fab-displaying phage libraries were screened by ELISA for their MET-binding and HGF-displacing abilities, and the positive clones were sequenced in the variable heavy (VH) and light (VL) regions. The VH and VL regions of the best-performing Fabs were fused to human constant heavy and light chain domains, generating 13 chimeric llama-human mAbs characterized by high-affinity MET binding and elevated HGF-displacing activity. The agonistic and antagonistic activities of the 13 chimeric antibodies were investigated in biochemical and biological assays and led to the identification of two antibodies, WT46 and WT52, which combine minimal agonistic effect with robust inhibitory activity, and represent good candidates for future application in anti-cancer therapy. Along with their ability to hamper HGF/MET signaling in a number of biochemical and biological assays, these antibodies displayed anti-tumor and/or anti-metastatic activity in preclinical models of HGF-dependent glioblastoma, triple-negative breast cancer, and colorectal cancer. Epitope mapping of the chimeric antibodies revealed that they bind to four different sites in the extracellular region of MET, two in the SEMA and two in the PSI–IPT domains. Since all antibodies were selected on the basis of their ability to compete with HGF binding to MET, this finding suggests that all four target hotspots identified are involved in the complex interaction between MET and HGF. ARGX-111, the final optimized antibody deriving from this series, is characterized by improved tissue penetration and enhanced ADCC, which result in increased anti-tumor and anti-metastatic activity in mouse xenograft models of both HGF-dependent and HGF-independent tumors (TAT—Targeted anticancer Therapies—2014 abstract [105]). It is currently under clinical evaluation in a Phase I trial in patients with advanced cancers that over-express the MET receptor (see Table 1).

Inhibition of the HGF/MET pathway may be also achieved by HGF-targeting antibodies, which sequester the ligand thereby preventing receptor activation. This approach, however, presents some bias: first of all, HGF is stored as an inactive precursor in the extracellular matrix of tissues, which represents an almost unlimited source of this factor. Furthermore, MET activation in cancer is often ligand-independent, deriving from genetic lesions as activating mutations, gene amplification, or post-transcriptional modifications. However, HGF derived from the tumor microenvironment has recently been shown to protect MET-amplified cells, otherwise sensitive to MET inhibitors, suggesting a role for the ligand in the resistance to anti-MET therapy, and providing the basis for the use of HGF-neutralizing drugs [106]. Finally, HGF plays an important role on tumor microenvironment, e.g., acting on MET-expressing endothelial cells and thereby stimulating tumor angiogenesis.

A number of anti-HGF antibodies have been generated. Among them, rilotumumab [107] and ficlatuzumab [108] are the best, characterized and are currently under clinical evaluation. Exhaustive information on anti-HGF therapeutic antibodies may be found in review [48].

The finding that MET can be activated through extensive cross-talk with other ligand/receptor couples [30,31,32,33,34] is emerging as a likely source of drug resistance and thus can be a limit to the therapeutic use of monoclonal antibodies against MET in the clinic; this should be taken into consideration. Indeed, in many ongoing trials another molecule, namely EGFR, is targeted alongside MET in a combined therapy (see Table 1) [109]. In this context, bispecific antibodies targeting two receptors were found to be more efficacious [110,111]. In support of this therapeutic strategy based on mAbs, recently a tetraspecific antibody recognizing EGFR, HER2, HER3, and VEGF was reported not only to inhibit signaling mediated by these receptors *in vitro* and *in vivo*, but, unexpectedly, to also disrupt HER–MET crosstalk [112].

In the context of cancer therapeutic applications, the same general disadvantages reported for other mAbs are present also in the case of anti-MET mAbs. Because of intrinsic bivalent Ab structure and the possibility of a mAb exerting both agonist—although only partial—and antagonist activity depending on the amount used, the amount administered *in vivo* is critical and must be carefully evaluated. On the other hand, their relatively short half-life *in vivo*, as well as the possibility that they can be rapidly and extensively consumed *in vivo* both by tumor and host cells [113] through their extensive internalization, must be taken into consideration and can explain their limited therapeutic activity despite optimized treatment regimens. Moreover, the therapeutic effect can also be affected by poor antibody penetration into the tumor. Fragmented engineered Abs are endowed with a higher penetrating ability in solid tumors, but this property is generally counterbalanced by their decreased half-life. As already discussed, one strategy for overcoming these disadvantages is coupling mAbs and their derivatives with molecules such as polyethylene glycol, which increase their half-life. A further improvement in this context is the coupling of mAbs to nanoparticles, in which they can act as targeting moieties, directing payload nanoparticles to the tumor sites (see next paragraph for more details).

## 7. mAbs as Targeting Moieties for Nanoparticle-Mediated Drug Delivery

Nanomedicine is a new field of medicine that exploits the properties of nanosized materials. In particular, nanoparticles have revitalized the ancient concept advanced by Paul Erlich of the “magic bullet” that can specifically target tumor cells. Indeed, because of their nanosize and thus their high surface/volume ratio and high surface bioreactivity, nanoparticles can be viewed as efficient multifunctional platform carriers for drug delivery and imaging probes and, if functionalized with probes for tumor markers, can be directed to the desired target [114,115,116,117,118]. Other advantages linked to their nanoscale dimensions are their prolonged circulation in the blood stream, since they can escape capture from phagocytic cells, and their accumulation at tumor sites, since they can cross the more permeable endothelial barrier present there [119,120,121]. This strategy was pursued with both a scFv anti-MET antibody linked to pegylated liposomes [122] and with the DO-24 mAb herein described, which was coupled to hydroxyapatite nanocrystals [123]. These nanocarriers were efficiently internalized within MET-expressing cells, where they released Doxorubicin, which was then translocated to the nucleus and exerted cytotoxicity [123]. Nanoparticle labeling with either fluorescein isothiocyanate [124] or quantum dots [122] allowed for tracking their interaction with cells and following their fate. Single-chain variable fragment (scFv) anti-MET antibody-pegylated liposomes were also shown to display anti-tumor activity “*in vivo*” [122]. This approach of active tumor targeting could represent an advantageous therapeutic strategy, since improved drug pharmacodynamics and pharmacokinetics, therapeutic index, and reduced side effects can be achieved.

## 8. Conclusions and Future Perspectives

The HGF/MET system can act as a double-edged sword, promoting both beneficial biological responses, as in the case of organ development during embryogenesis and tissue homeostasis in adults, and detrimental effects, as in the case of the uncontrolled cell proliferation of tumors. Monoclonal antibodies, which are extremely robust, specific, and versatile reagents, have achieved considerable success in cancer therapy over the past 15 years. MET-targeting antibodies endowed with antagonistic activities are currently under clinical evaluation in a variety of tumors, and appear to be promising tools for therapy. One of the limits of receptor tyrosine kinase (RTK)-targeted monotherapies, whether for antibodies or small molecules, is that only patients carrying a specific genetic lesion are suitable for the treatment, and even in the presence of the appropriate target not all patients respond to the therapy (primary resistance); furthermore, responding patients invariably develop resistance following treatment (secondary resistance). Combined targeting of different receptors, for example MET and EGF, the use of multitargeting inhibitors, or the association of targeted therapy and chemotherapy are promising strategies that are currently under clinical evaluation. Bispecific antibodies, simultaneously blocking multiple targets, represent a new and interesting tool [125].

Conversely, anti-MET antibodies inducing only some of the pleiotropic effects mediated by the receptor make feasible their use as agonists in the appropriate contexts, as in liver and kidney regeneration or myocardial protection. Their ability to induce partial MET activation, thereby evading HGF-induced undesirable activities, together with their enhanced stability, provide partial agonistic MET antibodies with great potential for application in regenerative medicine; more effort should be put into this relatively new field.
